# Simultaneous Application of Fibrous Piezoresistive Sensors for Compression and Traction Detection in Glass Laminate Composites

**DOI:** 10.3390/s111009478

**Published:** 2011-10-10

**Authors:** Saad Nauman, Irina Cristian, Vladan Koncar

**Affiliations:** 1 Institute of Space Technology, Islamabad 44000, Pakistan; E-Mail: saad.nauman@ist.edu.pk; 2 Technical University Gheorghe Asachi of Iasi, Iasi 700050, Romania; 3 ENSAIT, GEMTEX, Roubaix F-59100, France; E-Mail: vladan.koncar@ensait.fr; 4 University Lille Nord de France, F-59000, Lille, France

**Keywords:** intelligent sensors, intelligent structures, interconnected systems, monitoring, resistance measurement

## Abstract

This article describes further development of a novel Non Destructive Evaluation (NDE) approach described in one of our previous papers. Here these sensors have been used for the first time as a Piecewise Continuous System (PCS), which means that they are not only capable of following the deformation pattern but can also detect distinctive fracture events. In order to characterize the simultaneous compression and traction response of these sensors, multilayer glass laminate composite samples were prepared for 3-point bending tests. The laminate sample consisted of five layers of plain woven glass fabrics placed one over another. The sensors were placed at two strategic locations during the lay-up process so as to follow traction and compression separately. The reinforcements were then impregnated in epoxy resin and later subjected to 3-point bending tests. An appropriate data treatment and recording device has also been developed and used for simultaneous data acquisition from the two sensors. The results obtained, under standard testing conditions have shown that our textile fibrous sensors can not only be used for simultaneous detection of compression and traction in composite parts for on-line structural health monitoring but their sensitivity and carefully chosen location inside the composite ensures that each fracture event is indicated in real time by the output signal of the sensor.

## 1. Introduction

High performance composites with woven reinforcements have found wide applications in various industrial areas such as aerospace, aircraft, automobile, civil engineering, *etc*. Among other factors, reinforcement characteristics profoundly influence the mechanical properties of composites. For the manufacture of laminated composites, 2D woven reinforcements are mostly used when better mechanical properties are required in two dimensions (warp and weft directions). The crimp and fibre volume fractions can be adjusted in the warp and weft directions so as to design the reinforcement according to the stress-strain conditions. These so called 2D reinforcements offer certain important advantages such as easy and rapid production using conventional weaving technology and good in plane properties. These composites have found wide application in automotive, aerospace and civil engineering structures. In order to keep these structures operating safely and reliably it is important to incorporate some sort of monitoring system inside the structural component capable of providing real time, *in situ* information about the state or health of the component. In-service health monitoring of the structure can not only help in better understanding the deformation modes, but also in lowering the downtimes related to periodic maintenance. Moreover the record of stress-strain history prior to infliction of damage helps in understanding the cause of irreversible damage [1]. Different approaches that can be used for structural health monitoring (SHM) including ultrasonic scanning, acoustic emission (AE), shearography, stimulated infrared thermography (SIT), Fibre Bragg Grating (FBG) sensors, vibration testing, *etc*. have been discussed in detail elsewhere [2–5]. Today design engineers lay special emphasis on the integration of sensors during manufacturing process which enables them to perform *in situ* health monitoring of composite parts, reduce their cost and improve the accuracy of measurements. The classical NDE techniques hardly address this concern because of difficulties in *in situ* implementation.

A review of piezoresistive sensing methods already being applied to measure strain in fabrics/composites shows that several diverse sensing mechanisms exist. These approaches may be categorized on the basis of manufacturing technology as follows:

- Nanotube networks [6–11];
- Use of carbon tows for self-sensing [12–16];
- Semiconductive coatings [17–23].

None of these have gained universal acceptance either as standards in structural health monitoring of composites or for the fabrication of intelligent textiles. Nanotubes have been investigated in detail for use as sensing mechanisms, both for smart textile applications and for structural health monitoring of composites. Significant challenges still exist in their development, for example the efficient growth of macroscopic-length carbon nanotubes, controlled growth of nanotubes on desired substrates, durability of nanotube based sensors and actuators, effective dispersion in polymer matrices and their orientation. Therefore, there is a need to develop both experimental and analytical techniques to bridge the nano and macro scales towards optimization so as to use nanotube networks as sensors inside macroscale (fabric) or mesoscale (tow) composites.

Carbon fibre reinforced composites offer a unique possibility of using carbon tows as a sensing network because of their conductivity. The disadvantage of such an approach is that it can only be used for conductive fibre based composites. Moreover, it is imperative to understand the deformation mechanism of the reinforcement. Any anomaly in the deformation mechanism can threaten the sensing mechanism’s validity and efficacy.

As for semiconductive coatings, to date they have only been used for design of active components of intelligent textile structures, such as silicon flexible skins with regular textiles [24], flexible fibrous transistors [25–27] and other smart textile applications to manufacture consumer products and to detect physiological condition of the wearer [28–33].

It was suggested in our previous research work that the use of the intelligent textile approach in order to realize fibrous sensors compatible with SHM and composite technology [34] is a very promising solution for *in situ* health monitoring of composite parts. In the case of high performance textile composites these intelligent textile materials can be integrated during the manufacturing phase of the reinforcement or during the lay-up process. These materials perform dual functions inside a composite, as after integration in the reinforcement they not only act as an integral part of the structure, but also have actuating, sensing and microprocessing capabilities.

The piezoresistive sensor in fibrous form that has been developed is based on previous research studies on carbon black nanoparticles dispersed in polymers to form composites [17]. It has been successfully integrated into 3D-interlock carbon composite structural parts [34]. Measurements during tensile loading have shown promising results and it was concluded that such a sensor may be used as strain gauge inside composite structural parts for *in situ* online health monitoring applications. However, the theory of percolation indicates that the tensile deformation is not the only mode that contributes to the formation and deformation of percolation networks which influence the global conductivity [17]. The compression of piezoresistive conductive polymer composites used for coating yarns is also supposed to modify their conductivity by modification of internal conductive paths by orienting these paths in parallel or serial configuration and therefore altering the local resistance. These two phenomena—formation/deformation of conductive paths in traction and modification of the configuration of conductive paths in compression—are in competition. In some cases the first phenomenon is predominant, for instance when a sensor is used solely in tensile loading, while in the case of pressure or compression sensing, the second phenomenon is supposed to be predominant.

In this article, this dual sensing nature of our fibrous sensors is used in order to simultaneously detect two different deformation modes in a composite. This has been made possible by choosing different sensor locations inside the composite part. For instance, in case of a 3-point bending test it is possible to place one sensor near the surface in contact with the top bending support where compression is the main mode of deformation while the other sensor is placed close to the farther end (close to the stationary supports) of the composite part where traction is supposed to be the predominant deformation mode. These experiments have demonstrated the validity of a novel concept of structural health monitoring, not only capable of detecting traction and compression but distinctive fracture events inside the composite as well.

In order to perform bending tests, five-layer glass fibre laminate samples were prepared. Two sensors were placed between 1st and 2nd and 4th and 5th layers. These sensors were placed just under the top and just above the bottom layers so that the sensors were supposed to follow the deformation pattern of two distinctive composite faces corresponding to compression for the upper sensor and traction for the lower one. Afterwards, these specimens were tested for bending employing a 3-point bending test method [35]. Several different tests were performed in order to characterize the sensor response to various test configurations and fracture events.

The main objective was to determine the continuous deformation pattern of composite structures during bending, as well as to get information about discrete fracture events provoked by internal damages such as delamination, tow pullout and cracks at resin-tow interfaces, which correspond to “*hybrid measurements inside composites*”. The concept of hybrid measurement used in this paper is based on the approach developed in our previous studies on Piecewise Continuous Systems (PCS). The PCS has two time scales; the first time scale is continuous and the second one is discrete. Therefore such a system has two different functioning modes: continuous and discrete. The discrete functioning mode is relative to autonomous switching impulses. These theoretical bases have been used to develop novel control theories, but they can also be used in adaptive system configurations in order to develop a plant identification technique [36–42]. In fact, PCS are characterized by exogenous switching of their state.

The measurement results prove the PCS nature of the sensors developed in our laboratory. The discrete functioning mode may be observed when the slope of the measured signal changes, indicating that some internal structural change (delamination or crack propagation) occurred in the composite part. The constant slope indicates the proper continuous functioning mode for deformation in the same structural part. In terms of system approach this may be modeled as switching between different systems which correspond to different deformation modes. The switching moment, *i.e.*, a change in signal slope implies occurrence of internal structural changes due to particular fracture events. Therefore, our approach with sensors at two different levels can not only be used to detect deformation in composites, but also to detect internal changes that are often critical and may indicate irreversible damage in the composite structure.

## 2. Theoretical Section

**Figure 1.**
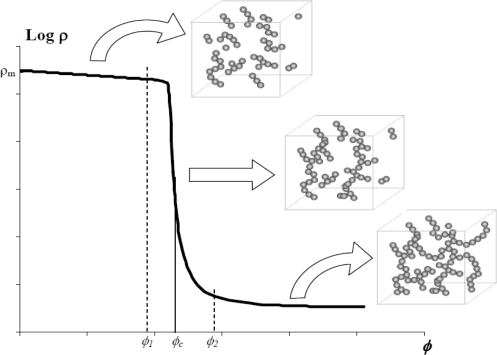
Evolution of electrical resistivity in a composite with volume concentration of electric fillers.

Let us consider a non-conductive polymer matrix charged with conductive fillers. Figure 1 shows the evolution of logarithm of resistivity “*ρ*” with volume fraction of conductive fillers “*ϕ*”.

The resistivity-volume fraction plot can be divided into two distinctive zones:

1. When the composite is an insulator. This is because of the fact that only a few charges are in contact which gives rise to few conductive networks for the flow of electrons in the composite.
2. When the composite is a conductor. Complete conduction arises from abundant filler contacts creating a conductive network for the flow of electrons and thus the electric current.

The transition between the insulating and conductive states occurs at a particular volume concentration of conductive filler. This critical concentration is termed as “percolation threshold”. Around the percolation threshold a sudden and rapid drop in resistivity is observed. This is because of the formation of conductive networks of fillers. At the percolation threshold these networks are just enough to allow electrons to flow through.

**Figure 2.**
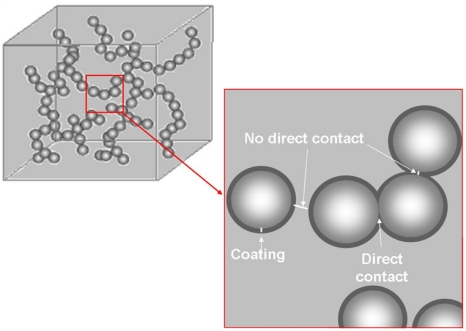
Schematic representation of filler contacts.

Conduction in such composites charged with conductive fillers depends on various phenomena, depending upon the filler geometry and their distribution in the polymer. Figure 2 shows that either fillers are in direct contact or they are separated by a layer of polymer matrix.

When filler particles are in direct contact, the electrical conduction is explained by metallic conduction and hopping. In metallic conduction the band structure of the material is overlapping which allows the electrons to flow from one site to another without energy input. When there is gap or barrier (a polymer layer in this case) between the filler particles, the electrons need to jump from one site to another. This hopping can be either

▪ Short range hopping: sites energetically distant and geographically close
▪ Variable range hopping: sites energetically close and geographically distant

**Figure 3.**
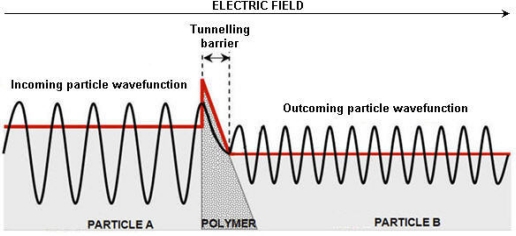
Schematic representation of quantum tunneling.

Due to its dual nature, as predicted by quantum mechanics, the electron is able to hop even though its kinetic energy is less than the potential energy of the barrier. The Heisenberg uncertainty principle suggests that an electron has a nonzero probability of moving from one side of any physical barrier to another. When an electron wave meets a potential barrier (polymer film), the wave does not instantly go to zero. Instead it starts to decay exponentially within the potential barrier. If the wave does not reach zero by the time it reaches the other end of the barrier then there is a finite probability that it will be found on the other side of the barrier implying that the wave effectively “tunnels” through the non-conductive barrier [43]. This is shown schematically in the Figure 3.

When these composites are subjected to tensile loading the filler particles displace relative to one another. This results in an average increase in inter-particle distance, breakage of certain percolation networks and an increase in potential energy of barriers for electron hopping. The combined effect of all of these phenomena is a net increase in the resistivity of the composite. This property can be used for detecting tensile loading in any composite part.

When compression is applied on a polymer composite filled with conductive particles, the inter particle distance is reduced. This translates into an increase in conductivity as more electrons can flow through filler particles physically in contact and tunnel through the barriers which under compression become “thinner” and the probability that decaying electron wave crosses over to the other end is higher. This property can be used for compression sensing.

## 3. Experimental Section

### 3.1. Sensor Design and Optimization

As coating solution, the conductive polymer composite based on dispersion of carbon black particles (Printex^®^ L6) in polymer (Evoprene^®^ 007) solution, using chloroform as a solvent was chosen [17]. In order to characterize the sensitivity and adherence of the coating on different substrates, the 35% carbon black solution was coated on different yarns (71 tex cotton spun yarns; 48.2 tex polyethylene monofilament and 25 tex polyamide monofilament). Visual inspection of the surfaces of coated yarns shows that the coating is more uniform for synthetic monofilaments compared to cotton yarns. The cotton yarns absorb the conductive solution, which penetrates inside the pores and interstices much like a dye. This particular phenomenon could be a source of non homogeneity in sensor electrical and mechanical properties, as the spun yarn is non uniform as compared to filaments, the coating and thus the resistivity achieved could be non uniform. Moreover the greatest inconvenience with coated cotton spun yarns is their low sensitivity during the initial tensile loading phase.

**Figure 4.**
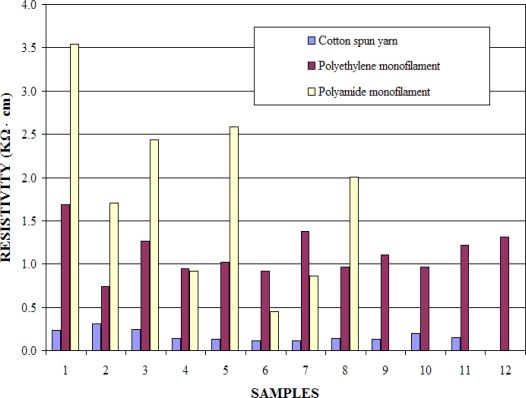
Resistivity values calculated for different substrates coated with 35 wt.-% carbon black solution.

The electrical resistance values were measured on 12 coated samples of each variant using a multimeter. The resistivities were then calculated using the yarn/filament fineness, yarn/filament lengths and these measured electrical resistance values. Figure 4 gives a comparison of calculated resistivity values of conductive layer deposited on different fibrous substrates. It can be seen that coated polyethylene filaments show relatively lower dispersion of resistivity as compared to coated polyamide filaments.

**Figure 5.**
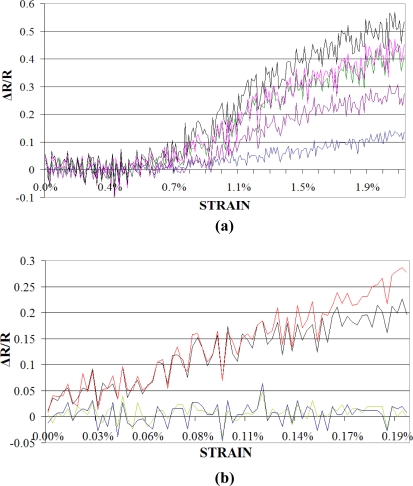

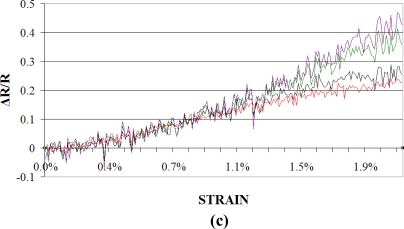
Electrical resistance variation during tensile strength tests on different yarn and filament substrates coated with 35 wt.-% carbon black solution. **(a)** Cotton spun yarns; **(b)** Polyamide monofilaments; **(c)** Polyethylene monofilaments.

In order to carry out tensile tests on coated yarns and monofilaments, an MTS 1/2 tester was used. Samples underwent quasi-static tensile loading at a constant test speed of 5 mm/min. For the purpose of electrical resistance variation measurement during the tensile testing, a simple voltage divider circuit and Keithley^®^ KUSB-3100 data acquisition module were employed. Figure 5 shows some of the results for electrical resistance variation, expressed as normalised resistance (ΔR/R) during tensile testing, obtained using different substrates for coating.

Initial electrical resistance of the coating on cotton yarns is much lower than on monofilaments, but since the cotton spun yarns are inherently irregular, the coatings obtained are not homogenous and the results for different coated yarns vary widely in their response to tensile loading [Figure 5(a)]. Due to particular fineness of the polyamide monofilament it was found that slight non-homogeneity in coating on the surface can result in breakdown of the conductive paths, as is obvious from Figure 5(b). As a result, the behaviour of polyamide is highly inconsistent. Polyethylene monofilaments provide a reasonably good compromise as the substrate. The coatings on polyethylene are easy to achieve due to good substrate/conductive solution interfacial properties. Moreover the relatively large diameter of the polyethylene monofilaments employed here, allow the substrate take up to be just enough for achieving measurable initial reistance of the coated sensors. As the curves in Figure 5(c) show, the polyethylene coatings are reproducible as the curves for all the four samples are nearly identical as opposed to polyamide and cotton. This is because of the fact that the coating achieved on polyethylene monofilaments are realtively homogeneous. Homogeneuous coatings result in lower dispersion of resistivity, as is obvious from curves in Figure 5(c). Another important feature of the curves presented in Figure 5 is the greater deviations in normalized resistance plots at higher strains. This again depends on the extent of uniformity achieved while coating the substrate. At higher strain rates, non uniform coatings tend to crack where there is a thin deposited layer. This causes a marked increase in resistance whenever a conductive track breaks up. Polyethylene substrates exhibit better performance in this respect, as well as the deviations at higher strains are minimal for curves presented in Figure 5(c). In view of all of these advantages, polyethylene monofilaments were chosen for sensor development.

In order to reduce the initial electrical resistance two ply polyethylene filaments were used. These were coated with the polymer solution as described above. The two ends of the coated polyethylene filaments were additionally coated with silver paint in order to reduce contact resistance and fine copper wire was attached to the two ends with the help of this paint. In this way, secure connections were realized. Sensor structural and geometrical parameters along with initial electrical resistance are shown in Table 1.

Prepared in this way, the sensor with polyethylene substrate was tested again on the MTS ½ tester for optimisation and calibration purposes. This test was performed under quasi-static tensile loading conditions at a constant test speed of 5 mm/min. As is obvious from the curves presented in Figure 5, these piezoresistive sensors produce a very small percentage change in resistance in response to physical phenomena such as strain. Moreover the output signal has considerable noise. Therefore a special data linearization module containing a Wheatstone bridge and an amplifier was used to measure unknown variable resistance of the sensor as a function of output voltage. Wheatstone bridges offer an attractive solution for sensor applications as they are capable of measuring small resistance changes accurately. The same Keithley^®^ KUSB-3100 data acquisition module was employed for the purpose of measuring voltage variation during tensile testing.

**Figure 6.**
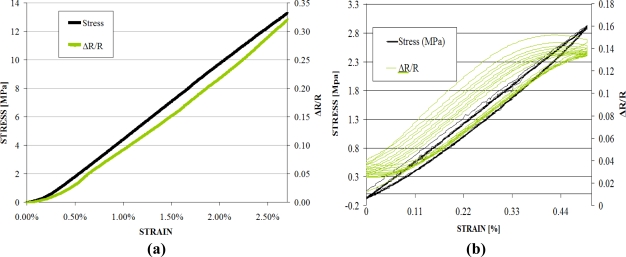
Normalized resistance and stress against strain for sensor outside composite. **(a)** Tensile test up to 2.75% elongation; **(b)** Hysteresis 10 cycles at 0.5% extension.

The resistance variation data thus obtained was treated for noise reduction using a low pass filter. The resultant stress-strain-resistance relationship curves for elongation of the out of composite sensor (before insertion in the reinforcement) are shown in Figure 6(a,b).

It may be noticed in Figure 6(a) that the stress *vs.* strain curve has the same shape as the normalised resistance (ΔR/R) *vs.* strain curve. This validates the electromechanical properties of our fibrous sensor for strains ranging from 0 to 2.75%. In Figure 6(b), the hysteresis results of the sensor for 10 cycles are given. For this test, the sensor underwent 0.5% extension at a constant cross head displacement rate of 5 mm/min, followed by compression in each cycle. The hysteresis test also shows that the sensor is capable of following the extension and compression patterns in each cycle. It can also be noticed that the hysteresis is high for the first cycle which reduces gradually and for the 10th cycle the sensor exhibits almost linear behaviour. This loss in hysteresis with increasing number of cycles can be attributed to permanent breakage of some of the percolation networks. It should be recalled that these sensors have been optmised so as to have volume concentration of carbon nanoparticles corresponding to the percolation threshold. At the percolation threshold the conductivity in the nano particle filled composites is due to particle-particle contact, electron hopping and tunnel effect. As the sensor undergoes repeated loading and unloading, some of the percolation networks responsible for conduction due to physical contact, break down completely. This results in sensor conductivity depending more and more on the tunnel effect with increasing number of cycles. This causes the sensor behaviour to become less hystyrical and more linear.

### 3.2. Composite Samples Preparation

Plain woven 2D fabric reinforcements were manufactured on a conventional weaving loom. Glass tows were used in warp and weft. Reinforcement characteristics have been summarized in Table 2.

**Figure 7.**
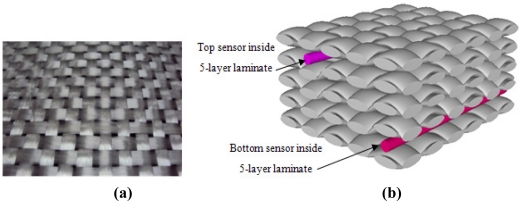
**(a)** Surface photograph of a plain woven 2D reinforcement; **(b)** TexGen generated geometry of 2D reinforcements placed over one another having two sensors at the top and bottom.

Afterwards five layers of these fabric reinforcements were placed over one another. The sensors were placed just under the 1st layer and just above the 5th layer during the lay up step so as to follow compression and traction loading respectively, during the 3-point bending test. A surface photograph of the plain woven 2D reinforcement is shown in Figure 7(a), while the TexGen [44] generated geometry of laminated composite specimen is shown in Figure 7(b).

**Figure 8.**
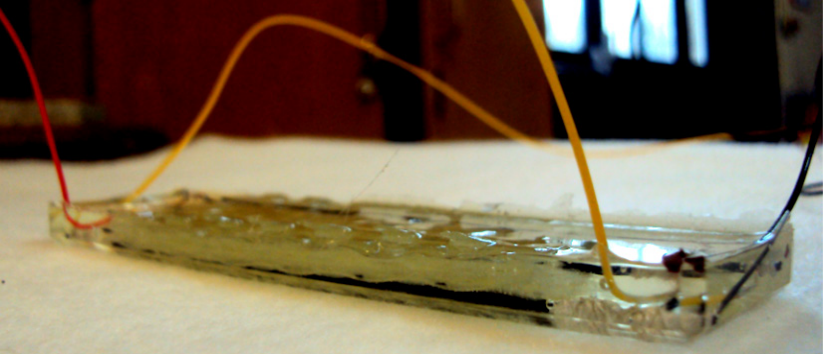
Glass laminated composite specimen with embedded sensors.

Afterwards the five-layer laminate structure with two sensors as described above was impregnated using a vacuum bag infusion process in order to make the composite part stiff. The resin employed was EPOLAM 5015 epoxy. The four connections of the top and bottom sensors which remain outside the reinforcement at the two ends were carefully separated from the rest of the mould. This was done by creating two vacuum sub-moulds inside the larger mould so that the resin would not impregnate the connections of the sensors. The impregnated composite samples were cut into slabs of required dimensions as shown in Figure 8. In this way each 12 cm × 3.0 cm glass composite specimen had two sensors, at the top and bottom, for compression and traction detection.

### 3.3. Data Acquisition Device

**Figure 9.**
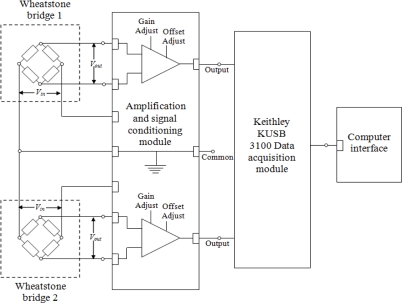
Schematic diagram of instrumentation amplifier and data acquisition module connected to sensors in Wheatstone bridge configuration.

A data amplification and signal conditioning module consisting of instrumentation amplifiers and the data acquisition module (Keithley^®^ KUSB 3100) were connected to the two sensors, in Wheatstone bridge configuration. This set-up is capable of accurately measuring small resistance changes. Figure 9 shows a schematic diagram of the data amplification and acquisition modules connected to the two sensors, in Wheatstone configuration, for simultaneous detection of compression and traction in the composite specimen.

## 4. Results and Discussion

### 4.1. 3-Point Bending Tests on Glass Laminates (until Fracture)

**Figure 10.**
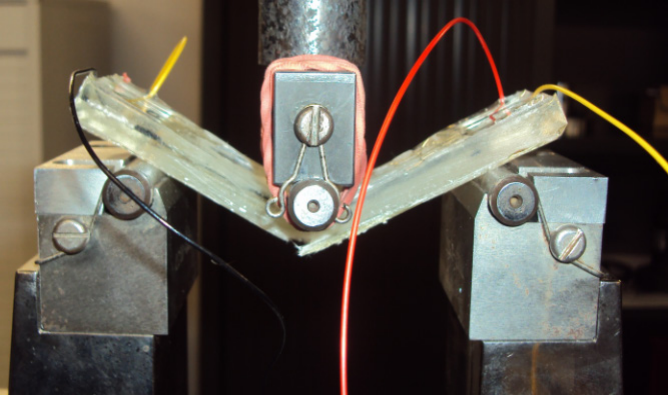
Glass laminate composite specimen with embedded sensors, loaded on an Instron 1185 tester for a 3-point bending test.

In Figure 10, a glass composite specimen can be seen loaded on an Instron 1185 tester during a 3-point bending test.

**Figure 11.**
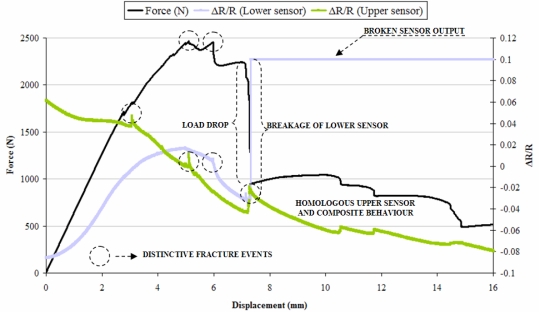
Force-displacement plot against normalized resistance variation for the two sensors inside laminated composite specimen tested until fracture at constant loading rate of 1 mm/min.

Composite specimens were loaded until fracture at a constant loading rate of 1 mm/min. A typical force-displacement plot against normalized resistance can be seen in Figure 11.

**Figure 12.**
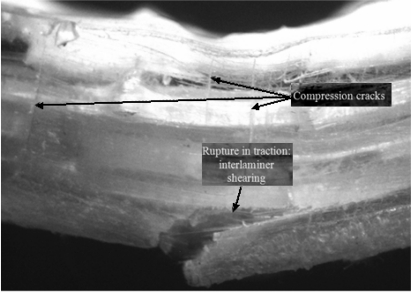
Transversal section of tested laminated composite specimen.

A photograph taken on transversal section of tested glass laminated composite specimens is shown in Figure 12.

A cursory observation of curves plotted in Figure 11 shows that the sensors are capable of following the loading and onset of damage in the composite. A sharp peak in upper sensor curve appeared much before the maximum load was achieved for the composite specimen. This might signify a distinctive fracture event such as a compression crack as the peak was accompanied by cracking sound and a slight change in the slope of Force-displacement plot as well. Compression cracks can be observed in Figure 12. The maximum load in force-displacement plot (Figure 11) coincides with a series of sharp peaks in the upper sensor curve. On the other hand the lower sensor curve starts dropping at the maximum loading point. The maximum load is followed by a sudden load drop which coincides with the lower sensor breakage as its output suddenly saturates at this point. An observation of broken laminated composite specimen photographs, given in Figure 12, reveals that the lower face of the composite has fractured with a certain degree of interlaminar shearing. This onset of interlaminar shearing might have caused a drop in the lower sensor curve (after the maximum loading), followed by complete breakage of the sensor due to fracture of the lower layers of the composite in traction. The maximum load drop in the force-displacement plot (Figure 11), which is conjectured to coincide with fracture in traction of the lower composite face, does cause a sharp peak in the upper sensor curve but is unable to cause its fracture. This is because of the fact that the interlaminar shear in the lower face does not advance transversally to cause complete breakdown of all the composite layers. This maximum load drop in the composite specimen is followed by a region where the load keeps on dropping gradually in steps. The most interesting aspect of this region is that the load drop is followed almost identically by the upper sensor.

### 4.2. Multyciclic Bending Test

**Figure 13.**
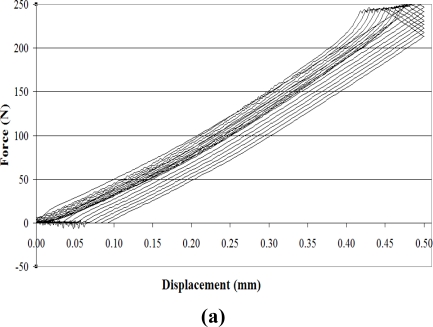

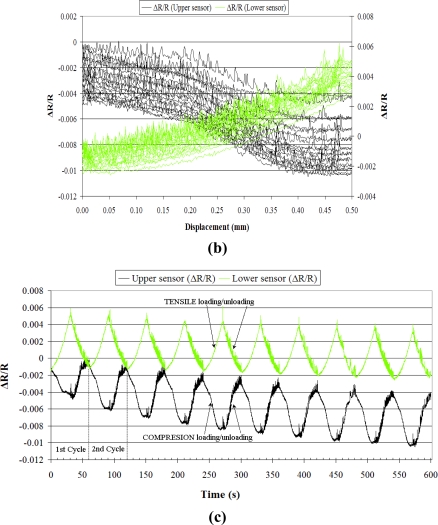
Multicyclic 3-point bending test of glass laminated composite **(a)** Force-displacement plot; **(b)** Normalized resistance curves for the two sensors against displacement; **(c)** Normalized resistance curve for the two sensors against time.

A multicyclic bending test was performed on the glass composite specimens. The tests were carried out at constant loading rate of 1 mm/min and consisted of 10 cycles. Maximum displacement of 0.5 mm was achieved during the course of each loading cycle. Force-displacement plots for this test are shown separately in Figure 13(a). Normalized resistance output for the two sensors against displacement in mm is given in Figure 13(b). The normalized resistance output for the two sensors is plotted in Figure 13(c) against time for clearer observation of sensor behaviour.

It can be observed from the force-displacement plot in Figure 13(a) that the composite exhibits certain hysteresis during cyclic loading. This hysteresis appears in the normalized resistance plots in Figure 13(b) and Figure 13(c) as well. In these two plots the hysteresis originates from composite as well as from the sensor behavior. Moreover the normalized resistance plot against time given in Figure 13(c) shows that the sensor signal is noisier during the unloading phase for both the upper and lower sensors. This can be attributed to phenomena occuring at the resin-sensor interface. It can be noticed that the unloading curves during tensile testing of “unintegrated sensor” given in Figure 6(b) don’t show any noise. Therefore it can deduced that the noise during unloading of “integrated sensors”, as can be seen in Figure 13(c) is due to the resin-sensor interface or the 3-point bending test fixture itself. During unloading the rigid matrix interface does not tend to regain its initial dimensions as does the elastic sensor. Inside the composite, the sensor is thus “constrained” by the matrix. This “disparity” in sensor behaviour and its environment appears as noise in the unloading plots.

The noise associated with unloading plots can also arise from the test fixture itself. Unlike tensile testing in which the two ends of the specimen are gripped by jaws, in 3-point bending tests, the specimen is not gripped at all. During loading the specimen is positively held in between the three points. This causes a smooth signal, as is evident from Figure 13(c). While unloading causes relaxation of the specimen placed between the three points. This relaxation causes vibrations in the specimen, generating noise, which is evident from the unloading plots in Figure 13(c). This conjecture is also supported by the fact that the noise in signal output increases as the unloading decreases from 0.5 mm (maximum strain) displacement to 0 mm displacement (complete unloading), so that the noise is maximum for complete unloading in each of the cycles.

A difference in amplitude of the two sensor signals, as is obvious from “tensile” and “compression” plots given in Figure 13(c), can be attributed to two factors:

▪ The two sensors don’t have equal initial resistance. This is because of the manual coating technique adopted for their fabrication. Due to lack of automation in the layer deposition process, identical sensors cannot be produced. The initial resistances of the two sensors (Table 1) varies slightly from batch to batch.
▪ The gain used for the amplification of the two sensor signal outputs is not exactly identical. This is because of the fact that the gain of each amplifier is regulated by a potentiometer whose resistance is varied using a manual set screw. Thus it is virtually impossible to fix identical gain for the two amplifiers.

When a comparison is made between the two plots, it is revealed that the upper sensor curves appear to be noisier and more hysterical as compared to the lower sensor curves. This signifies an important difference in energy absorption/release mechanism and mechanical deformation phenomena (compression and traction) at the top and bottom faces of the composite specimen during cyclic loading.

**Figure 14.**
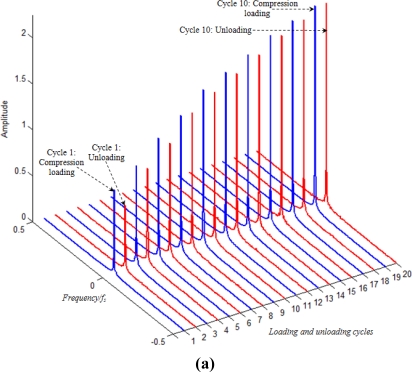

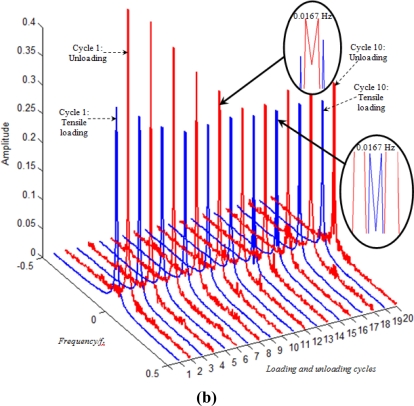
FFT analysis on **(a)** upper sensor signal during cycling loading and **(b)** lower sensor signal during cycling loading.

FFT (Fast Fourier Transformation) analysis of the normalized resistance signals for upper and lower sensors during Multicyclic loading test performed on laminated glass composite specimens [Figure 13(c)] is given in Figures 14(a,b), respectively.

Cyclic tests were carried out on glass laminate samples in order to characterize the sensor performance during loading and unloading of the composite specimen. Loading corresponds to tension on the lower face and compression on the surface of the specimen. It was observed that during the loading phase of each cycle the signal had different form as compared to that during unloading. In order to analyze and quantify sensor performance, FFT was applied to the complete sensor signal output. For each traction or compression cycle, FFT was applied separately to the loading and unloading phases. Thus in Figure 14, the z-axis represents the number of loading and unloading cycles (in compression for the upper sensor and in traction for the lower sensor). The plot corresponding to 1 on z-axis thus represents loading phase in 1st cycle, whereas the 2nd plot corresponding to 2 on z-axis is representative of the frequency distribution during the unloading phase of the 1st cycle. In this way there are 20 FFT plots in each of Figures 14(a,b) corresponding to loading and unloading during 10 cycles for the upper and lower sensors, respectively. In Figures 14(a,b) the amplitude of FFT is plotted on the y-axis, whereas the x-axis represents the FFT frequency from 0 to 0.5 × f_s_ Hz, where f_s_ is sampling frequency, equal to 10 Hz.

Each loading or unloading phase in a cycle corresponds to 300 data points. Therefore the FFT was applied on 300 data points. For most of the FFT plots the frequency of loading and unloading cycles (0.0167 Hz) can be observed. This frequency appears as dual “crater” like peaks in the FFT plots. Some of these peaks are shown enclosed in ellipses in Figure 14(b). Indeed the magnitude of these peaks is higher then the magnitude of noise that appears in FFT plots representing unloading.

Complete absence of measurement noise that can originate from forming and deforming of percolation networks during tensile and compression loading and unloading phases can also be observed. This is because of the fact that our sampling frequency (10 Hz) was rather low for the observation of such high frequency phenomena. It can also be observed that the loading plots are less noisy as compared to unloading plots. It might be conjectured that this noise is generated by the visco-elastic behavior of the sensor and composite during the unloading phase (red plots in Figure 14(a,b)).

## 5. Conclusions

A new system for real time *in situ* health monitoring of structural deformations in composites has been developed and applied in this article. Fibrous sensors developed as piecewise continuous systems (PCS) which can not only measure compression and traction, but are also capable of detecting distinctive fracture events in a composite structure, have been successfully integrated into a composite laminate during the lay-up phase. The sensors provide complete stress-strain history of the composite specimen. The 3-point bending test results given in this paper have proven the feasibility of our approach. These results indicate that very useful information on composite “health” and about the phenomena occurring inside the structure under quasi-static loading conditions, related to crack propagation, fracture, delamination and other disturbances can be extracted. However, the data thus generated will have to be properly treated in order to extract all the supplementary information unavailable until now. Such treatment techniques, adaptable to composite structures are currently under development in our laboratory.

Moreover, the sensors will have to be improved and optimized, particularly in terms of their bandwidth, sensitivity and compatibility with carbon and other high performance multifilament tows that are being widely used to design fibrous reinforcements for high performance composite structures. As a next step of this research work, these sensors will be integrated to more advanced 3D composites for the detection of distinctive fracture events. This will allow us to develop a pool of information about the deformation behaviour of different types of composites during different loading conditions.

## Figures and Tables

**Table 1.** Sensor properties.

**Parameter**	**Value**
Average linear density of the filament (g/km)	48.23
Average diameter of the filament (mm)	0.7
Average width of the sensor cross section (mm)	1.68
Average thickness of the sensor cross section (mm)	1.26
Aspect ratio of the sensor (width/thickness)	1.33
Length of the sensors (cm)	11.7
Initial resistance of upper sensor (kΩ)	34
Initial resistance of lower sensor (kΩ)	24

**Table 2.** Reinforcement and composite specifications.

**Parameter**	**Value**
Linear density of warp tow (tex)	2331
Linear density of weft tow (tex)	2331
Average thickness of reinforcement (mm)	0.8
Warp tows density (tows/cm)	18
Weft tows density (tows/cm)	16
Areal weight (g/m^2^)	789
Fiber volume fraction (%)	60

## References

[1] Wang S, Chung DDL (2006). Self-sensing of flexural strain and damage in carbon fiber polymer-matrix composite by electrical resistance measurement. Carbon.

[2] Black S Structural Health Monitoring: Composites Get Smart.

[3] Farrar CR, Worden K (2007). An introduction to structural health monitoring. Philos. Trans. A Math. Phys. Eng. Sci.

[4] Balageas D, Balageas D, Fritzen CP, Guemes A (2006). Introduction to Structural Health Monitoring. Structural Health Monitoring.

[5] Chung DDL (2002). Composites get smart. Mater. Today.

[6] Hecht DS, Liangbing H, George G (2007). Electronic properties of carbon nanotube/fabric composites. Curr. Appl. Phys.

[7] Thostenson ET, Chou TW (2008). Carbon nanotube-based health monitoring of mechanically fastened composite joints. Compos. Sci. Technol.

[8] Li C, Chou TW (2008). Modeling of damage sensing in fiber composites using carbon nanotube networks. Compos. Sci. Technol.

[9] Kang IP, Schulz MJ, Kim JH, Shanov V (2006). A carbon nanotube strain sensor for Structural Health Monitoring. Smart Mater. Struct.

[10] Zhao H, Zhang Y, Bradford PD, Zhou Q, Jia Q, Yuan FG, Zhu Y (2010). Carbon nanotube yarn strain sensors. Nanotechnology.

[11] Lee SS, Lee JH, Park IK, Song SJ, Choi MY (2006). Structural health monitoring based on electrical impedance of a carbon nanotube neuron. Key Eng Mater.

[12] De Baere I, van Paepegem W, Degrieck J (2010). Electrical resistance measurement for in situ monitoring of fatigue of carbon fabric composites. Int. J. Fatigue.

[13] Kaddour AS, Al-Salehi FAR, Al-Hassani STS, Hinton MJ (1994). Electrical resistance measurement technique for detecting failure in CFRP materials at high strain rates. Compos. Sci. Technol.

[14] Muto N, Arai Y, Shin SG, Matsubara H, Yanagida H, Sugita M, Nakatsuji T (2001). Hybrid composites with self-diagnosing function for preventing fatal fracture. Compos. Sci. Technol.

[15] Abry JK, Choi YK, Chateauminois A, Dalloz B, Giraud G, Salvia M (2001). *In-situ* monitoring of damage in CFRP laminates by means of AC and DC measurements. Compos. Sci. Technol.

[16] Kupke M, Schulte K, Schüler R (2001). Non-destructive testing of FRP by D.C. and A.C. electrical methods. Compos. Sci. Technol.

[17] Cochrane C, Koncar V, Lewandowski M, Dufour C (2007). Design and development of a flexible strain sensor for textile structures based on a conductive polymer composite. Sensors.

[18] Zhang W, Blackburn RS, Dehghani-Sanij AA (2009). Carbon black reinforced epoxy resin nanocomposites as bending sensors. J. Compos. Mater.

[19] Dittmar A, Meffre R, Oliveira FD, Gehin C, Delhomme G Wearable Medical Devices using Textile and Flexible Technologies for Ambulatory Monitoring.

[20] Huang C-T, Shen C-L, Tang C-F, Chang S-H (2008). A wearable yarn-based piezo-resistive sensor. Sens. Actuat. A: Phys.

[21] Huang C-T, Tang C-F, Lee M-C, Chang S-H (2008). Parametric design of yarn-based piezoresistive sensors for smart textiles. Sens. Actuat. A: Phys.

[22] De Rossi D, Carpi F, Lorussi F, Paradiso R, Scilingo EP, Tognetti A (2005). Electroactive fabrics and wearable man-machine interfaces. Wearable Electronics and Photonics.

[23] Scilingo EP, Lorussi F, Mazzoldi A, De Rossi D (2003). Strain-sensing fabrics for wearable kinaesthetic-like systems. IEEE Sens. J.

[24] Katragadda RB, Xu Y (2008). A novel intelligent textile technology based on silicon flexible skins. Sens. Actuator A: Phys.

[25] Lee JB, Subramanian V (2005). Weave patterned organic transistors on fiber for E-textiles. IEEE Trans. Electr. Devices.

[26] Guerra EM, Silva GR, Mulato M (2009). Extended gate field effect transistor using V_2_O_5_ xerogel sensing membrane by sol-gel method. Solid State Sci.

[27] Choi MC, Kim Y, Ha CS (2008). Polymers for flexible displays: From material selection to device applications. Prog. Polym. Sci.

[28] Carpy F, De Rossi D, Lorussi F, Mazzoldi A, Scilingo EP, Tognetti A (2002). Electroactive fabrics for distributed, conformable and interactive systems. IEEE Sens. J.

[29] Wijesiriwardana R, Mukhopadhyay S, Mitcham K Knitted Strain Gauges.

[30] Wijesiriwardana R (2006). Inductive fiber-meshed strain and displacement transducers for respiratory measuring systems and motion capturing systems. IEEE Sens. J.

[31] Baurley SL Smart Textiles for Future Intelligent Consumer Products.

[32] Kim S, Leonhardt S, Zimmermann N, Kranen P, Kensche D, Muller E, Quix C Influence of Contact Pressure and Moisture on the Signal Quality of a Newly Developed Textile ECG Sensor Shirt.

[33] Koncar V, Kim B, Nebor EB, Joppin X FICC (Floatable Intelligent and Communicative Clothing) Project Conductive Fibers Development.

[34] Nauman S, Lapeyronnie P, Cristian I, Boussu F, Koncar V (2011). On line measurement of structural deformations in composites. IEEE Sens. J.

[35] NF EN ISO 14125 (1998). 1998—Composite plastiques renforcés de fibres—Détermination des propriétés de flexion: Conditions d'essai pour les composites plastiques renforcés de fibres par la méthode de chargement en trois points ou en quatre points. CEN.

[36] Koncar V, Vasseur C Control of Linear Systems Using Piecewise Continuous Systems.

[37] Chamroo A, Vasseur C, Koncar V HAOPI: Hybrid Adaptive Online Plant Identification.

[38] Chamroo A, Vasseur C, Koncar V (2007). A piecewise continuous approach for on-line identification of continuous plants. Control Eng. Appl. Inform.

[39] Wang H, Vasseur C, Koncar V, Chamroo A Hybrid Control for Vision Based Cart-Inverted Pendulum System.

[40] Wang H, Vasseur C, Koncar V Friction Compensation of an X-Y Robot Using a Recursive Model Free Controller.

[41] Wang H, Vasseur C, Koncar V, Christov N Recursive Model Free Controller used in MIMO Nonlinear System’s Trajectory Tracking.

[42] Wang H, Vasseur C, Koncar V, Christov N Trajectory Tracking of Vision Based Robot Systems Using Piecewise Continuous Controllers and Observers.

[43] Peratch, QTC Science http://www.peratech.com/qtcscience.php.

[44] TexGen Software www.nottingham.ac.uk/~emxmns/texgen.htm.

